# Improving Inpatient Endocrine Referral Management Using the Alertive App and a Structured Registrar Rota: A Quality Improvement Project at a UK District General Hospital

**DOI:** 10.7759/cureus.87794

**Published:** 2025-07-12

**Authors:** Husam Jamil, Daniel McNally, Preet Mukesh Shah

**Affiliations:** 1 Acute Internal Medicine, Health Education Yorkshire and Humber, Wakefield, GBR; 2 Acute Internal Medicine, Mid Yorkshire Teaching NHS Trust, Wakefield, GBR; 3 Diabetes and Endocrinology, Mid Yorkshire Teaching NHS Trust, Wakefield, GBR

**Keywords:** endocrinology and diabetes, mobile app, pdsa cycle, quality improvement project (qip), referrals

## Abstract

Disorganization in inpatient referral management can be due to multiple reasons and causes delays in patient care. We assessed the use of a hospital-approved mobile app called Alertive for referral management. The use of the app improved the referral handling process and led to the formulation of a structured rota for the first time, representing a sustainable change. Our project showed that the use of secure apps can be the future of inpatient referral management.

## Introduction

The management of inpatient endocrine referrals within the current endocrine team at this UK district general hospital was disorderly. Various issues surrounded this aspect, including the absence of a focused referral method, a lack of awareness among teams regarding how and whom to refer to, and a lack of organization among registrars (middle grade doctors responsible for responding to referrals) in assuming the referral role for the day between hours of 9 am and 5 pm. 

We conducted a retrospective analysis of data for our quality improvement project (QIP). Since this was a QIP rather than a clinical audit, we commenced our journey of improvement without the use of any specific guidelines or standards, as it is commonly understood that improving referral systems leads to timely patient reviews and, hence, better patient outcomes [1,2]. 

We aimed to achieve timeliness and efficiency in managing inpatient endocrine referrals by the use of the Alertive app. We planned data collection to measure improvement, and the changes implemented included a timely review, either as a face-to-face consultation or advice, by endocrine registrars within 24 hours of receiving referrals. We also aimed to enhance the use of the Alertive app by increasing awareness among referring teams about the app and the introduction of the first-ever registrar rota. We aimed to achieve these targets through Plan-Do-Study-Act (PDSA) cycles, performing a minimum of two cycles. The duration of this QIP was six months. 

In this report, we discuss a QIP in which we attempted to improve the referral system to the endocrinology team via a trust-approved app for referral response called "Alertive."

## Materials and methods

Sample

All inpatient referrals via the Alertive app were included in this QIP, and the data were analyzed retrospectively. The first PDSA cycle data gathering was done from September 8, 2023, to October 6, 2023. The data gathering for the second PDSA cycle was done from November 10, 2023, to December 8, 2023. Total referrals were 58 during these days, but two patients were not included from PDSA1 as they were referred during nationwide junior doctor strike days in the UK. Twenty-two of these were from the first PDSA cycle and thirty-four from the second, giving a total of N=56. Inclusion and exclusion criteria are made evident.

Inclusion and exclusion criteria

The inclusion criteria for the study consisted of inpatient referrals made exclusively through the Alertive app. Referrals were excluded if they occurred on strike days or if they involved patients who were not classified as inpatients.

The rationale behind this decision was to establish an improved referral system specifically for inpatient referrals, as this mode of referral constituted the majority of cases to endocrine registrars, where the disorganization was most prominent. Incorporating data from strike days would probably have reflected subpar performance, given the unavailability of doctors during those periods. It is worth mentioning that collecting data on the volume of referrals made prior to implementing the app was very difficult, as referrals were made as direct phone calls to registrars. Also, training programs in the UK are mainly rotational in nature, meaning registrars are not permanent staff in the endocrinology department. Therefore, collecting pre-intervention metrics was ethically problematic. The hospital provided training on the use of the Alertive app. The aim was reviewed by advice or face-to-face within 24 hours of the referral being made. The time was calculated as per the timings written on the sent and received messages in the app, which are time-stamped. The data was collected manually. The ward round would start with the consultant (senior most doctor) who would ensure that there is a registrar holding the Alertive role as per the rota, and if not, he would either designate it to another registrar or the on-call consultant would assume the role. Project approval was obtained from both the hospital as well as the endocrine department. 

Alertive app

Alertive was an application procured by the Trust (hospital) to replace bleeps. It allows secure messaging via clinicians' own mobile devices or via computer applications. This software is able to handle direct messaging, task management, and critical alerting. This has now replaced all bleeps, including crash team membership roles, and has been successfully implemented into many departments of the hospital. Devices can connect to Alertive through the trust’s secure Wi-Fi, with very positive feedback from users, decreasing the number of devices doctors have to carry and reducing the insecure use of social media applications. The Alertive app was used to calculate time durations between the referral made and the referral response by endocrine registrars. The Alertive app is General Data Protection Regulation (GDPR) approved. 

Rota design

The first step involved assessing clinic and ward rosters to fairly distribute working days among available registrars. Allocating referral days to those with minimal clinic commitments was preferred, though not always possible. Challenges arose, especially when only one registrar was available for the entire department, and managing the schedule became hectic. Despite challenges, the current roster system is an improvement over the previous bleep system. It ensures that secretaries are informed about the designated contact person for referrals through the distribution of a roster copy.

**Figure 1:**
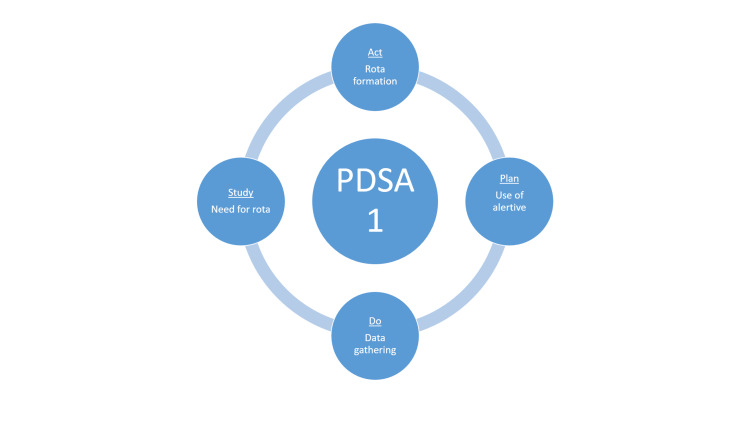
PDSA1 cycle studied the disorganisation in managing referrals and concluded that forming a rota was important, which was then implemented PDSA: Plan-Do-Study-Act Image credit: Husam Jamil

**Figure 2:**
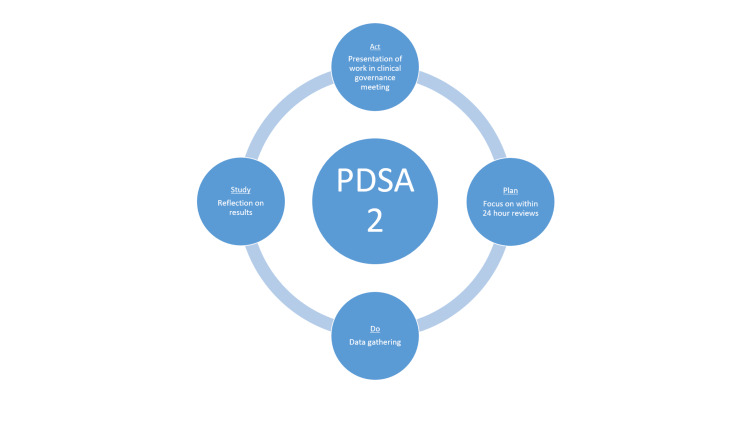
PDSA2 cycle focused on data gathering through the app. Achievements were noted and discussed in the clinical governance meeting PDSA: Plan-Do-Study-Act Image credit: Husam Jamil

The Microsoft Excel sheet (Microsoft Corp., Redmond, USA) was created for data collection. All graphs and charts were made on Microsoft Excel. Two PDSA cycles were employed (Figure 1 and Figure 2). 

The "Plan" phase of PDSA cycle 1 included steps to enhance the use of the Alertive app. This involved responding to anyone who called the endocrine registrar to discuss a referral by directing them to use the Alertive app instead, after which the conversation about the patient continued on the app. Additionally, the endocrine secretaries were emailed and instructed to redirect anyone who contacted them for registrar phone numbers to Alertive for inpatient review requests. The "Do" phase involved data gathering from September to October 2023. The "Study" phase included reflection on the data, which strongly indicated the need for a rota due to some delayed reviews. The "Act" phase involved proposing and approving the formation of a rota and emailing all specialty doctors to encourage more frequent use of the Alertive app.

**Figure 3:**
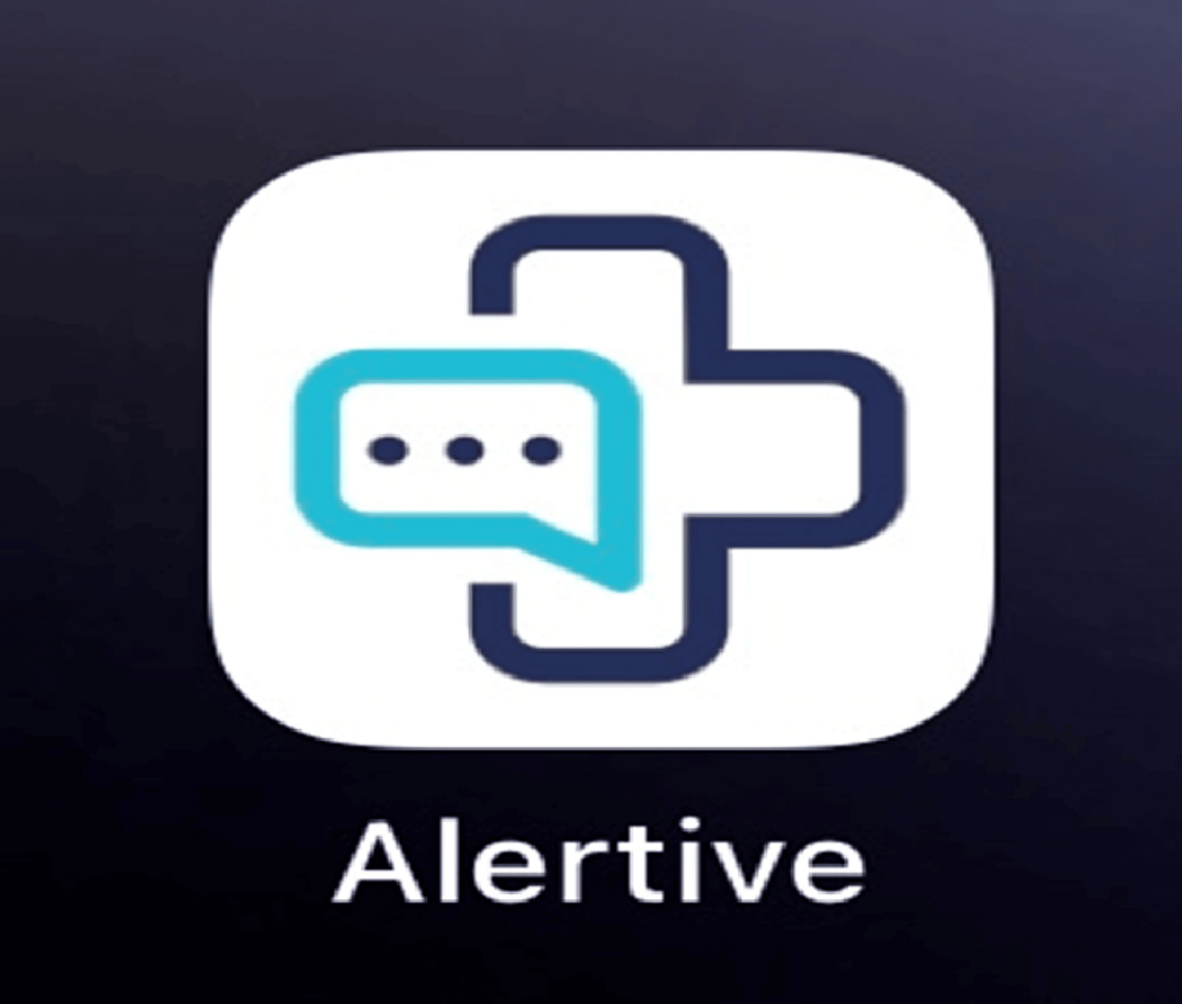
Screenshot image of the Alertive app icon

The "Plan" phase of PDSA cycle 2 focused on encouraging all registrars to perform same-day reviews. The "Do" phase involved data gathering from the Alertive app. The "Study" phase included reflections on both achievements and areas for ongoing improvement. The "Act" phase involved presenting the findings at the clinical governance meeting. Data collection from the Alertive app (Figure 3) was straightforward, as all conversations, along with the time taken by registrars to provide advice on referrals, were automatically saved on the platform.

## Results

**Figure 4:**
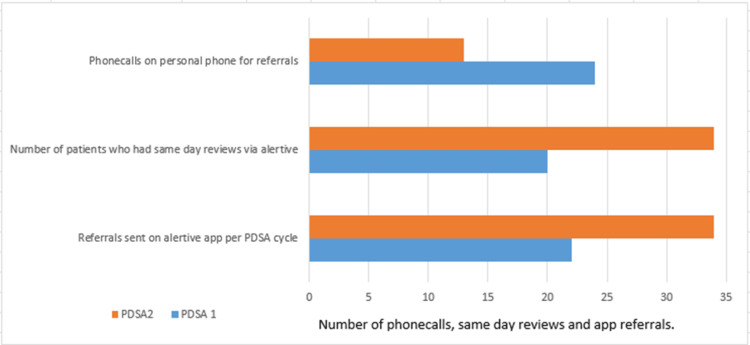
A bar chart showing improvement in PDSA2 on various parameters PDSA: Plan-Do-Study-Act

Bar charts, tables, and run charts were formulated once the data was finalized. Our QIP showed an overall improvement in successfully increasing the referrals via Alertive for inpatient queries (54.55%), reducing phone calls to the private phones of registrars (45.83%), increasing the number of patients who had endocrine reviews on the same day of referral by 10%, and improving the median time to review by 28.57% in PDSA2 compared to PDSA1 (Table 1 and Figure 4). The number of referrals varied considerably throughout the weeks of PDSA cycles, and the average number of referrals was seven per week.

**Table 1:** Table showing improvement in same-day reviews, reduced phone calls, and rota availability for the organization among registrars PDSA: Plan-Do-Study-Act

Parameters measured for improvement	PDSA1	PDSA2	% improvement
Referrals sent on the Alertive app per PDSA cycle	22	34	54.55
Number of patients who had same-day reviews via Alertive	20	34	10
Phone calls on personal phone for referrals	24	13	45.83
Rota availability for registrars for referral response	Nil	Available	100
Median time to referral response by endocrine registrars	1.4 hours	1 hour	28.57

**Figure 5:**
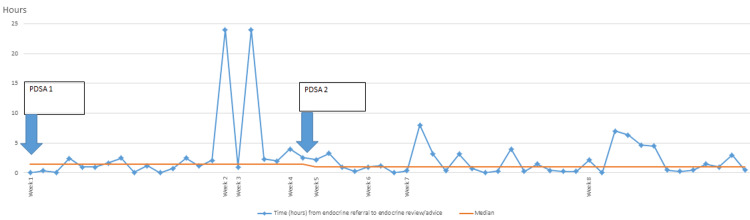
A run chart displaying the variability of review times by endocrine registrars over eight weeks. PDSA cycles 1 and 2 were implemented in weeks one and five, respectively. The median review time was 1.43 hours during PDSA1 and 1 hour during PDSA2 PDSA: Plan-Do-Study-Act

The run chart (Figure 5) provides key observations. The number of data runs appears sufficient for the data provided. Notably, there is a significant change in the median value starting from week five following the implementation of PDSA2, with the median time decreasing from 1.43 hours to 1 hour. Some instances in weeks two, four, seven, and eight show considerably higher values than the median, indicating outliers.

While no consistent trends or patterns are evident across the weeks, the shift in median suggests improvement in week five. Overall, the chart suggests an enhancement in the process, evident in the lower median time from week five onward and the absence of exceptionally high data points after the implementation of Improvement Plan 2. However, the presence of outliers and variability in the data suggests that, although the median time has improved, there may still be instances of prolonged review times, indicating the need for further refinement.

**Figure 6:**
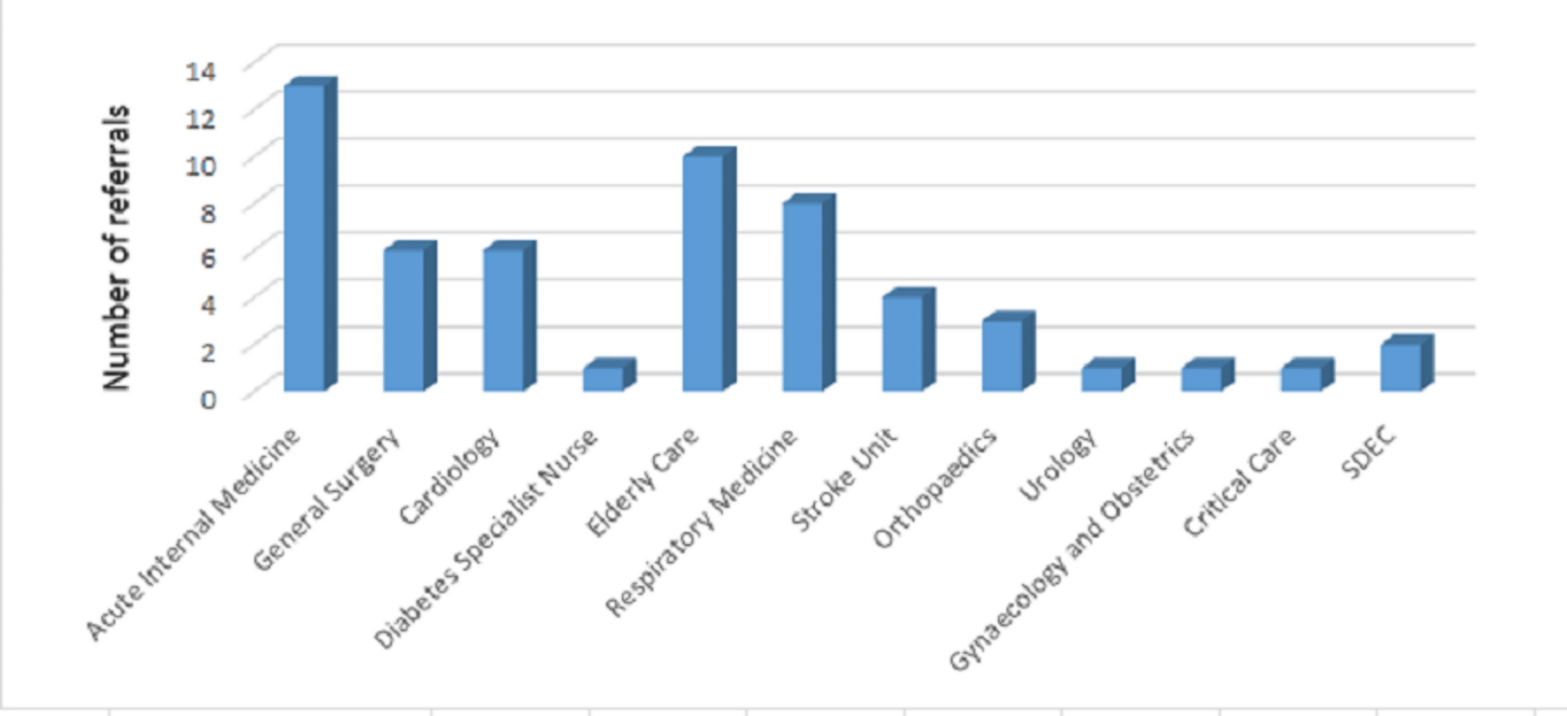
A bar chart displaying the number of referrals per adult inpatient team SDEC: same day emergency care

Most inpatient referrals (Figure 6) out of the total 56 referrals came from the specialty of Acute Internal Medicine 23.21% (n=13), Elderly care 17.86% (n=10), and Respiratory medicine 14.29% (n=8). Cardiology and General Surgery referrals were both 10.71% (n=6). The Stroke Unit made 7.14% (n=4) of the referrals, Orthopedics made 5.35% (n=3), same day emergency care (SDEC) made 3.57% (n=2), and 1.79% (n=1) each were from Urology, Gynecology and Obstetrics, Diabetes Specialist Nurses, and Critical Care.

**Figure 7:**
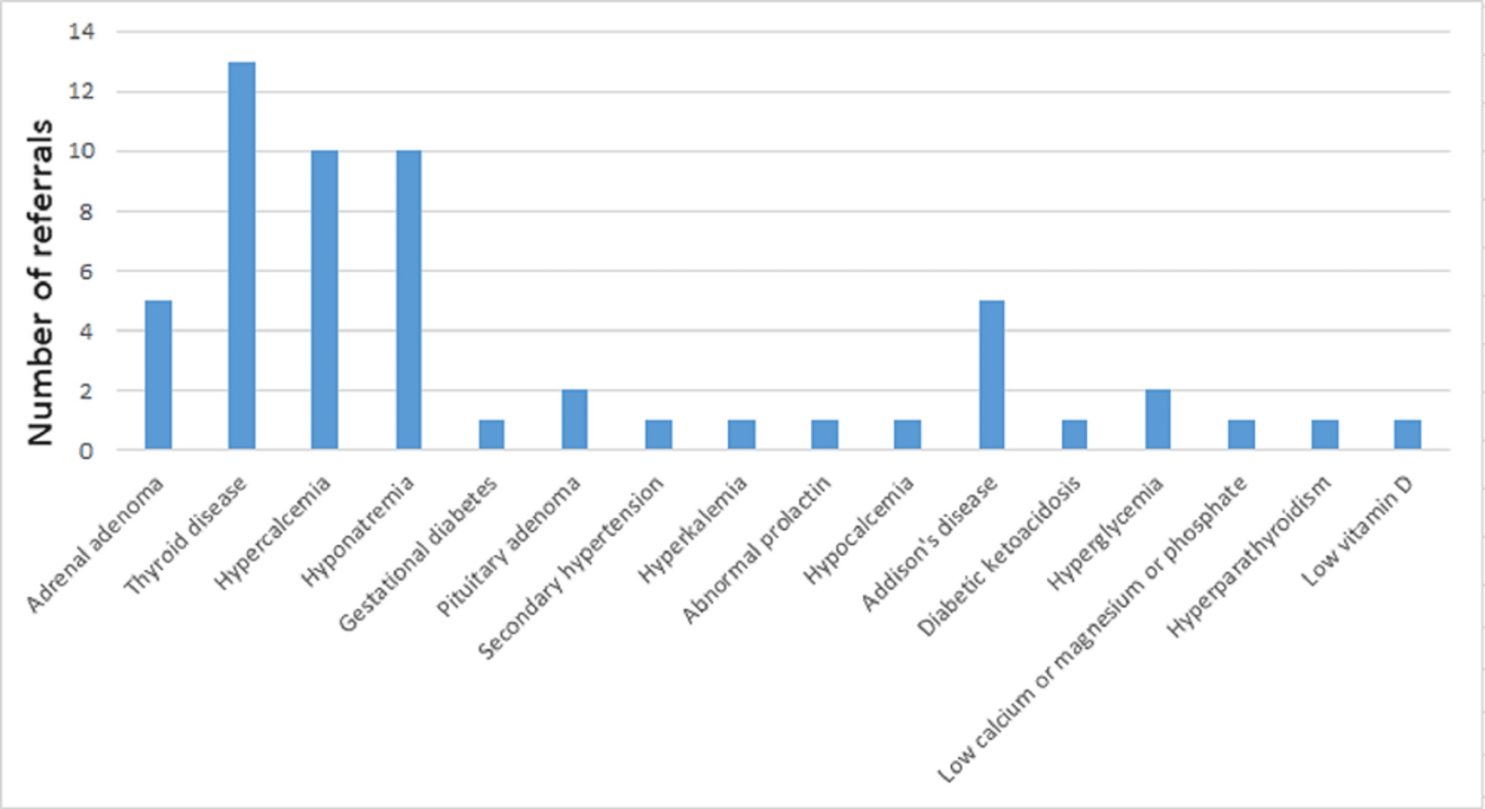
A bar chart showing clinical questions asked of the endocrinology team by other specialties to aid patient management

The most commonly referred endocrine problem (Figure 7) was thyroid disease, accounting for 23.21% (n=13), followed by hypercalcemia and hyponatremia at 17.86% (n=10). Adrenal adenoma and Addison’s disease accounted for 8.93% (n=5), and pituitary adenoma and hyperglycemia for 3.57% (n=2). Conditions such as gestational diabetes, hypertension, hyperkalemia, abnormal prolactin, hypocalcemia, diabetic ketoacidosis, low calcium, magnesium, or phosphate, and low vitamin D each accounted for 1.79% (n=1).

## Discussion

Prior to our project, referrals to the Endocrinology team at this hospital were made through emails to secretaries, phone calls to registrars on their personal mobile phones, and occasionally, phone calls to consultants. Numerous incidents arose due to these practices. For example, phone calls were often made to registrars when they were not at work, and the email trail was frequently incomplete or inadequately followed up. The endocrine team operates across three sites, and registrars were frequently occupied with clinic or ward duties at these locations, making it challenging to respond to referrals promptly. This disarray resulted in numerous concerns and dissatisfaction from referring teams.

Use of mobile apps in healthcare is on the rise, ranging from patient management to inpatient hospital referral management [3-5]. The Alertive app is a "WhatsApp-like" app that was secured by our hospital to replace bleeps and allows secure messaging, task management, and critical alerting, including crash bleeps, using secure Trust Wi-Fi. The feedback has been encouraging from the users, as there is now a decreased number of devices doctors have to carry. Use of this app for endocrine inpatient referral management was a game changer for improved management, as indicated by our QIP results. 

Our project was in line with other studies showing that the use of technology leads to better referral management [6]. The use of electronic referral systems not only streamlines patient flow and enhances organization but also increases doctors' morale and contributes to positive patient experiences [7]. Reduced miscommunication between sending and referring teams also optimizes patient care [8,9]. 

The knowledge base in diabetes and endocrinology is ever-increasing, and more evidence is emerging on how endocrine disorders can have deteriorating effects on multiple organs [10,11]. Hence, prompt response and treatment strategies suggested by the endocrine team for inpatient management are crucial for patients located in wards of other specialties. Our data (Figure 6) shows how diverse the referring teams can be. Hence, prompt responses to referrals can lead to reduced hospital stays of patients in other wards, and therefore, a good referral system is pivotal [12]. 

The project led to the rota implementation, which was a key achievement. Designing rotas was a challenge, but led to significant improvement in organization and same-day reviews. The registrar doctors on a typical day in our hospital have ward, clinic, and on-call commitments. The challenge in rota design was allocating the role to someone who is not in the clinic, and hence, any gaps were escalated to the responsible consultants. Gaps in the rota can potentially lead to harm to patients and lower doctor morale; hence, every effort was made to minimize gaps [13,14]. 

We used specific, measurable, achievable, realistic, and timely (SMART) criteria in our objective planning [15]. The SMART goals were to enable enhanced use of the Alertive app, ensure that inpatient reviews or advice by the endocrine team occurred within 24 hours of referral, and reduce phone calls made to registrars' personal phones by in-hospital teams.

Our project over a short span of six months showed notable outcomes. We enhanced the use of the Alertive app by 54%. Qualitatively, rota formation as a sustainable change and improved understanding of our fellow inpatient teams on the correct referral method led to more organization. The registrars received fewer phone calls on their phones. The strongest indicator of our success is the run chart (Figure 5), which has shown increased app use following PDSA2 and enhanced same-day reviews. We presented our work in the hospital's clinical governance meeting and received profound appreciation. 

There were many limitations to this QIP, and therefore, we ask that our findings be interpreted with caution. The sample size for the retrospective study was small and non-random, the data collection period was short, and the doctor strike days influenced the results. The data was collected manually, and changes in registrar behavior toward referral management were not measured. Larger, randomized studies are needed in the future to improve the generalizability and credibility of these findings.

## Conclusions

Our QIP, over a short span of six months, showed notable achievements. We enhanced the use of the Alertive app, and there were no reviews conducted later than 24 hours by endocrine registrars during the second PDSA cycle. The biggest success was the formulation of the registrar rota, which remarkably streamlined the referral review process by endocrine registrars. The process also led to a greater understanding among referring teams of the correct method of referral. Further PDSA cycles in this project could include improvements in referral processes between the hospital and primary care. More studies are needed in the future to explore the advantages and disadvantages of app use.
